# Demonstrating environmental impacts on the sound structure of languages: challenges and solutions

**DOI:** 10.3389/fpsyg.2023.1200463

**Published:** 2023-07-17

**Authors:** Ian Maddieson, Karl Benedict

**Affiliations:** ^1^Department of Linguistics, University of New Mexico, Albuquerque, NM, United States; ^2^College of University Libraries and Learning Sciences, University of New Mexico, Albuquerque, NM, United States

**Keywords:** language location, language structure, language and environment, global environmental data, geographic information systems, analysis platform

## Abstract

Recent research has suggested that there are significant associations between aspects of the phonological properties of languages and the locations in which they are spoken. In this paper we outline a strategy for assembling maximally reliable and well documented climatic and environmental data to place in juxtaposition with carefully curated linguistic information on both language location and structure. Problems with temperature records are specifically highlighted as an illustration of the use of the platform and considerations when selecting environmental data for analytic use. Preliminary analyses suggest that certain previously proposed language-environment relationships are statistically valid, but that these may be better placed in a broader framework of language types.

## 1. Introduction

In recent years there has been increasing interest in the hypothesis that some aspects of the phonological structure of spoken languages are shaped at least in part by ecological and climatic factors in the area in which they are spoken (Munroe et al., 1996; Fought et al., 2004; Everett, 2013, 2017; Everett et al., 2015, 2016; Maddieson and Coupé, 2015; Maddieson, 2018). There are several challenges in addressing this question and this paper is focused on considering how to respond to these challenges. We see these as essentially four inter-related issues:

1. How can potentially relevant ecological and climatic factors best be tracked over appropriate time periods and spatial scales given available data?
2. How can appropriate locations and boundaries be established for an individual language’s area over which relevant environmental variables will be defined?
3. How can similarities between languages due to inheritance be distinguished from possible effects of environmental conditions?
4. How can theoretically motivated correlations be distinguished from spurious ones?

In this paper we discuss approaches to these challenges and describe the development of publicly shared data and tools to address them. We consider the provision of these data and tools a major contribution of the current project.

These issues are also affected by which languages are included in a survey, what factors are used in their selection, and how their individual phonological properties are identified. We start with a discussion of the language sample we have compiled.

### 1.1. The language sample

Our sample of just over 1,000 languages, represents about 1/7th of “living languages” according to the categorization in the Ethnologue (Eberhard et al., 2022), that is, languages still currently spoken, or sufficiently well-documented while still in community use. The sample aims to meet multiple criteria. It includes representatives of all language families with 20 or more members in the Ethnologue listing, as well as many members of smaller families and isolates. It aims in part to reflect language density by selecting multiple languages from areas where many are spoken, mainly in tropical regions not far from the equator, but builds upon this sample by seeking to include languages spoken in the widest diversity of environments, including in desert and high-altitude locations and at high latitudes. These are regions with low language density and hence seeking to populate such areas in our sample results in the inclusion of some quite closely related languages, such as varieties in the Inuit and Saami stocks in northern latitudes, or languages found in hot desert regions in north Africa or south-western South America. In some cases, languages only recorded in documents dating as far back as the 18th century have been included to increase geographical diversity. However, inclusion of these languages is considered crucial since variables encoding altitude, temperature, vegetation type and seasonal variation have been put forward as influences on language structure, and some of these variables tend to exhibit lower variance in the areas near the equator where language density is greatest.

### 1.2. Assigning locations

Locations where languages are spoken are identified in different studies in one of two ways, either as points or as areas. The two major on-line catalogs of languages, Ethnologue (Eberhard et al., 2022) and Glottolog (Hammarström et al., 2022) take opposing sides on this issue. Ethnologue provides maps delineating areas for each language, whereas Glottolog provides a single point. There are several significant matters to consider. Although many languages have been spoken primarily in quite small, localized areas over relatively long time periods this is not the case for others. Some speaker populations are quite widely dispersed while others have moved from previous locations, either voluntarily or under duress.

**Figure 1:**
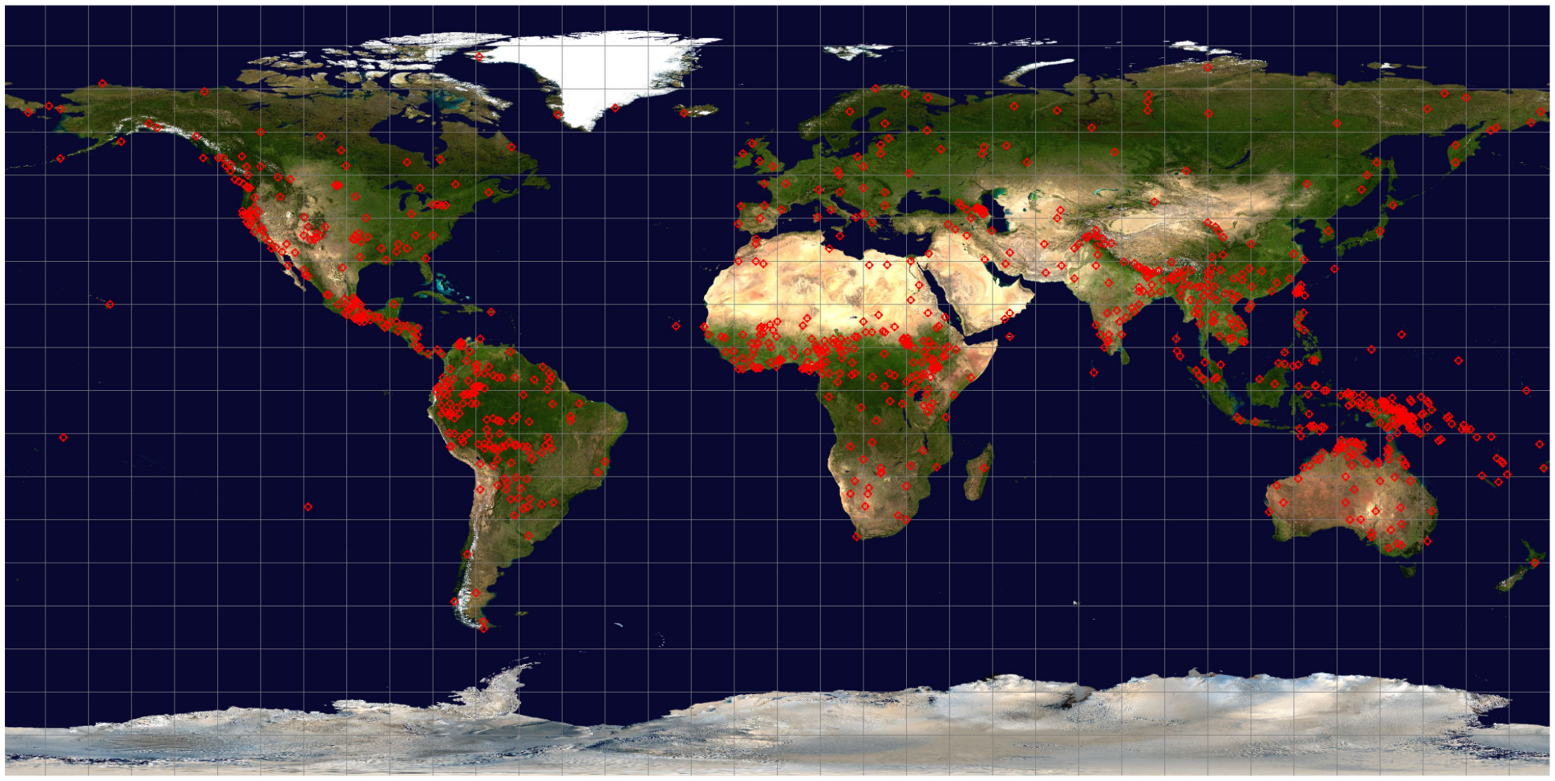
Global distribution of languages included in the dataset. Each language location is indicated by a red diamond superimposed on the Blue Marble Next Generation (NASA Earth Observatory, 2005) global satellite image.

We have adopted an approach that combines point and areal locations. A primary point location is chosen for each language, usually the main center where the current speaker population is found, the location where specific fieldwork was conducted for minority languages, or the primary political center for more widely spoken languages (e.g., Paris for French, Jakarta for Indonesian). Around this location a 100 km radius is established to encompass the terrain and climatic conditions in the area. To accommodate the proximity of competing languages in the locality, the point locations for all neighboring languages taken from Glottolog were obtained and Voronoi diagrams (Atsuyuki et al., 2000, p. 2) constructed around these locations. When environmental values within a given language’s vicinity are sampled, those values jointly within the established Voronoi cell and the 100 km radius are included. The point locations of the languages included in the project database are illustrated in Figure 1.

We also attempt to distinguish speaker populations that have remained in a given local area from those that have been displaced. If there are connections between climatic and environmental properties, these would be expected to be more evident in the subset of languages that have been spoken in the same location over an extended period of time, for our purposes set as at least an estimated 300 years. Stable languages include cases like the Berber language Siwi, spoken in an oasis in western Egypt as far back as records extend, as well as English, where basic characteristics of the standard language were established in London in the 17th century. Garifuna is an example of a ‘displaced’ language, since the present location of speakers in coastal Honduras and Belize dates only to the early 19th century.

### 1.3. Controlling for inheritance

Discussion of typological issues in linguistics must always consider whether cross-linguistic similarities are the result of shared (genealogical) inheritance or due to other factors, either linguistic or non-linguistic. A common approach in the past focused on constructing a language sample selected to maximize the independence of the languages chosen, e.g., by including only one member from any higher-level genetic grouping. A more recent trend has been to relax the criteria for inclusion and try to account for inherited similarity by using a statistical model that includes family membership as a control. There are several problems with this approach. One is that there are many languages that are isolates or belong to very small families, so that the degrees of freedom of this variable are very large if all families are included. Alternatively, isolates and small families may end up excluded from analysis; for example, Hay and Bauer (2007) exclude 44% of their sample when examining the extent to which phoneme inventory size is independent of family affiliation. Another problem is that there is no consensus on the membership of many of the larger language families. For example, Ethnologue (Eberhard et al., 2022) includes many groups of languages in families such as Australian, Nilo-Saharan, Niger-Congo and Trans-New Guinea that are excluded from the nearest equivalent ‘top-level’ families recognized in Glottolog (Hammarström et al., 2022). Campbell and Poser (2008, especially chapters 6 and 9) provide a very balanced discussion of the history of proposals for language family affiliations.

In our work we are trying a different approach, namely constructing a single scalar variable to represent degree of language relatedness. A value on this scale is attributed to each pair of languages in our sample. A value of 10 means that the language pair in question do not belong together in any language family that is widely accepted by experts. A language isolate will thus have the value 10 with all other languages. At the other end of the scale, a value of 1 represents two speech varieties that are considered by some to be dialects of a single language, as, for example, East and West Greenlandic. The value 9 is used where there are strongly divided opinions as to whether certain languages do or do not belong together in a highest-level family. This value is assigned, for example to Japanese and Korean with regard to languages in the Altaic family (itself quite widely disputed) as there is a substantial group of linguists who find support for their inclusion in a ‘Macro-Altaic’ or ‘Transeurasian’ family using traditional methodology (Georg et al., 1999; Robbeets and Savelyev, 2020), despite many skeptics. Other proposed macro-families, such as Nostratic (Bomhard, 2008) or Eurasiatic (Greenberg, 2000), are not considered at all plausible. Values 2–8 represent closer to more distant degrees of relationship within generally agreed-upon language families. These values are assigned based on two factors. The first is the internal branching structure of the language family as suggested in the compilations found in Ethnologue and Glottolog and compared to the most recent published studies on individual families or groups, such as Julian (2010) on Iroquoian, Ratliff (2010) on Hmong-Mien, Whiteley et al. (2018) on the Bantu subgroup of Niger-Congo, or Michael and Chousou-Polydorou (2019) on South American language families in general; language pairs that join at a higher branch of a tree are assigned a higher value than pairs that join at a lower level. The second factor reflects a judgment on the internal diversity of the family. In families with little internal diversity, reflecting an assumed shallow time depth, the highest node is assigned a lower value than in more diverse families. Thus, the most distant languages in the Quechuan and Witotoan families have the value 5, as these families are close-knit. The only pair of languages in our sample from the New Guinea Border (or Tami) family, Waris and Imonda, are assigned a value of 3. In more diverse families — the majority — the most distant pairs are assigned the value 8. These assignments are clearly somewhat imprecise, but we do not believe that any more exact alternative exists at present.^1^

**Figure 2:**
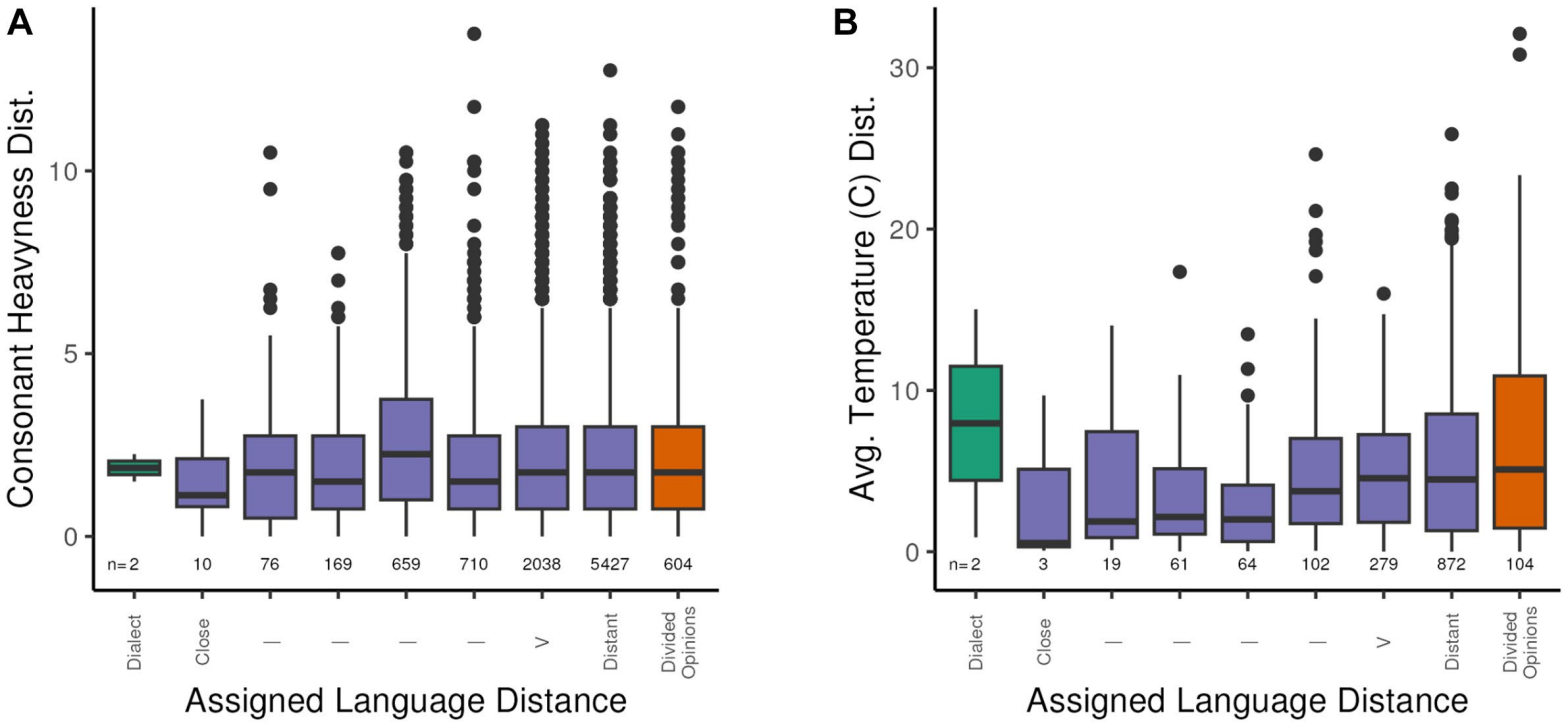
Pairwise differences in CHeavyLog **(A)** and Average Temperature **(B)** values between related languages by pairwise language distance labeled “Close” for closely related languages (distance value of 2 in source data) to “Distant” for distantly related languages (distance value of 8 in source data). The plot also includes potential dialects labeled as “Dialect” in the figure, and languages for which the relatedness is in dispute as “Divided Opinions” (see section 2.2.1 for a discussion of the reduced number of languages for which precipitation data are available).

A brief illustration of how these distances might be used is illustrated by Figure 2, which plots the pairwise distance between related pairs of languages (i.e., excluding those with the value 10) against the pairwise difference between the languages on the ConsHeavy variable (see Table 1 for definition) and the pairwise difference between languages for the Average Annual Average Temperature (v_tavg_dC__avg, see Table 2 for definition). The figure shows that increasing ‘genetic’ distance between languages does not correlate with greater pairwise difference in ConsHeavy (Figure 2A), while showing a slight stepwise increase in temperature variation with increased language pair distance (Figure 2B). In other words, more closely related languages are not any more similar to each other in consonant heaviness than more distantly related languages are. In contrast, there is a slight increase in average temperature as language pair distance increases, potentially related to increased geographic sample distances between less closely related languages.

**Table 1:** Language parameters.

Language parameter	Parameter name	Description	Values
Maximum onset	Onset	Index of onset complexity	0–3 (0 = single C onset)
Maximum coda	Coda	Index of coda complexity	0–3 (0 = no coda allowed)
Basic vowel qualities	VQ	Simple vowels only	2–20
Total number of vowels	VTotal	All vowels including long, nasalized, diphthongs, etc.	2–72
Vowel index	VowIndex	Proportion of vowel symbols in simplified transcriptions of short wordlists (from Everett, 2017)	0.251282051–0.646551724 (available only for a subset of our sample)
Number of consonants	CTotal	Total of consonants in inventory	6–128
Number of obstruents	Obstr	Total of obstruents in inventory	4–122
Sum of consonants and vowels	SegTot	Sum of VTotal and CTotal	11–156
Sum of consonants and basic vowels	CplusVQ	Sum of VQ and CTotal	11–133
Percentage of obstruents	ObsPct	% of obstruents in CTotal	17.3913–100
Complexity of tone system	ToneCat	Categorical labeling of tone systems	None, Marginal, Simple, Moderately Complex, Complex
Tone system index	ToneOrdinal	Rank order of tone system complexity	0–3 (0 = no tone; 3 = complex tone system)
Maximum onset and coda	OnsCoda	Sum of Onset and Coda	0–6
Consonant heaviness index	ConsHeavy	Sum of OnsCoda plus CTotal/4	1.2–33
Consonant heaviness index, Obstruents only	CHeavyObstr	Sum of OnsCoda plus Obstr/3	1.33* - 41.66*
Log-based consonant heaviness	CHeavyLog	Sum of OnsCoda plus (log)CTotal	1.7976–10.0604
Obstruent laterals	ObsLat	Presence/absence of /ŋ/ in inventory	Yes/No
Front rounded vowels	FRndV	Presence/absence of front rounded vowels	Yes/No
Glottalized consonants	GlotC	Presence/absence of glottalized consonants in inventory	No, Ejectives (Ej), Implosives (Imp), Resonants (Res), Ej & Imp, Ej & Res, Ej Imp & Res, Imp & Res, Plosives (Korean only)
Presence of ejectives	Ejectives	Presence/absence of ejectives in inventory	Yes/No
Number of ejectives	#Ejectives	Number of ejectives in inventory	0–19
Presence of implosives	Implosives	Number of implosives in inventory	Yes/No
Number of implosives	# Implosives	Presence/absence of implosives in inventory	0–6
Glottalized sonorants	GlotRes	Presence/absence of glottalized sonorants in inventory	Yes/No
Number of glottalized sonorants	#GlotRes	Number of glottalized sonorants in inventory	0–8
Velar nasal	VelarNas	Presence/absence of /ŋ/ in inventory	Yes/No
Nasalized vowel pattern	NVPattern	Nasalization contrast affecting basic vowel qualities	None, Some, All
Prenasalized consonants	PNC’s	Presence/absence of prenasalized stops in inventory	Yes/No
Vowel length pattern	VLength	Vowel length contrast affecting basic vowel qualities	None, Some, All, Other (more long than short vowels)
Aspirated stops	Aspirates	Presence/absence of aspirated stops or affricates	Yes/No

**Table 2:** Sample derived environmental variables and their associated descriptions, units and aggregation methods.

Environmental variable name	Description	Units	Aggregation Method
v_elev_m__median	Elevation	m	Median
v_qa_unitless__median	Specific Humidity	Unitless	Median
v_biomass_MgHa__median	Above ground live woody biomass	Mega-grams / Ha	Median
v_lc_tall_ct__sum	Tall vegetation land cover	Count	Total number of raster elements of this type
v_lc_med_ct__sum	Medium height land cover	Count	Total number of raster elements of this type
v_lc_short_ct__sum	Short land cover	Count	Total number of raster elements of this type
v_lc_water_ct__sum	Water land cover class	Count	Total number of raster elements of this type
v_lc_snow_ct__sum	Snow land cover class	Count	Total number of raster elements of this type
v_tavg_dC__avg	Average annual average temperature	°C	Average
v_tmax_dC__avg	Average annual maximum temperature	°C	Average
v_tmin_dC__avg	Average annual minimum temperature	°C	Average
v_prcp_mm__avg	Average annual precipitation	mm	Average

Aggregation methods describe the method used to calculate a single value from the multiple individual environmental parameter values within each language’s truncated Voronoi cell.

### 1.4. Specific hypotheses

The specific hypotheses that have guided the collection of data for our project link linguistic attributes to ambient temperature, precipitation, humidity, vegetation density and altitude. We briefly review the proposals that have been made.

Munroe et al. (1996), Munroe and Silander (1999), and Fought et al. (2004) proposed that a higher proportion of simple consonant-vowel (CV) syllables, that is, syllables with simply one consonant at the beginning and only a following vowel, was favored in warmer areas, defined as those with at least 5 months of the year in which mean monthly temperature did not fall below 10° C. CV frequency was counted in short wordlists of about 200 words from 53 languages. The rationale offered by these authors was that warmer climates lead to more time outdoors and more “distal communication.” Simple CV syllables are proposed as optimal for more distant speech since “the hearer benefits from perceptual distinctness, and the speaker, in conveying messages with these minimal syllabic units rather than more complex ones, achieves economy of articulation.” A balance between perceptual distinctiveness and economy of articulation is commonly assumed to be a fundamental essential of spoken language (Lindblom, 1990), so this argument amounts to saying that this balance requires a different equilibrium in outdoor versus indoor communication.

A rationale for such a difference can perhaps be found in Maddieson and Coupé (2015) and Maddieson (2018). These studies, inspired by the Acoustic Adaptation Hypothesis proposed especially by biologists studying birdsong (see Boncoraglio and Saino, 2007; Ey and Fischer, 2009 for reviews), proposed that degraded acoustic transmission conditions correlate with simpler signals. Maddieson and Coupé (2015) found that a quantity they named ‘consonant heaviness’, combining the overall size of the consonant inventory and the complexity of syllable onsets and codas, is lower in languages spoken in hotter, wetter, and more densely vegetated areas. Since temperature, precipitation and vegetation density are strongly positively inter-correlated, and correlate negatively with altitude and rugosity, they distilled these measures into a first principal component, rather than selecting one factor as the single explanans. This analysis used a sample of 633 languages. In a follow-up study, Maddieson (2018) measured the proportion of time in short spoken extracts from 100 languages (a subset of the 633) that is sonorant in character, i.e., consists of either vowels or voiced sonorant continuants like nasals or approximants. The resulting sonority index correlated highly with consonant heaviness. In this study mean annual temperature emerged quite clearly as the strongest predictor of sonority. Speech with a higher proportion of sonorants has more slowly modulated changes and higher temperatures are known to degrade more rapidly changing signals. Over time, languages used in warmer environments, especially outdoors might end up with simplified acoustic structures, probably due to hearers tending not to perceive more rapidly modulated aspects of the signals.

A related paper had been published around the same time by Everett (2017), proposing that languages in areas of higher specific humidity had a higher proportion of vowels in their lexical forms. He calculated a “vowel index” from short wordlists of over 4,000 ‘doculects’ included in the AJSP database as of 2016 (Wichmann et al., 2022). The vowel index is the total number of letters representing vowels in a given wordlist divided by the total number of letters representing consonants and vowels together in that wordlist, as represented in the simplified transcription used in the AJSP, which among other things ignores vowel length. The vowel index correlates highly with the consonant heaviness index of Maddieson and Coupé (2015), despite the fact that, as Everett rightly notes, measures such as the size of a consonant inventory do not reflect the relative frequency of use of the elements it contains. Everett argues that languages in dry regions exhibit a bias toward less vocal cord usage since desiccated air makes phonation a little more difficult. Vowels, being the proto-typical voiced sounds, would therefore be less frequently used in low-humidity areas. Everett also found an association between higher temperature and higher vowel index values, but a weaker one than that with humidity.

Everett et al. (2015, 2016) have also proposed an association between tone and high humidity based on similar reasoning. They argue that precise control of phonation frequency is more difficult in low humidity conditions, so tone contrasts, particularly more complex tone systems, tend to be avoided where ambient humidity is low. Since all languages use variations in fundamental frequency to encode critical information, the argument that tone is specifically liable to be affected by low humidity has been challenged (Ladd, 2016).

Everett (2013) posited that the inclusion of ejectives in a language’s consonant inventory is favored if the language is spoken at or near high altitude. Two rationales are proposed; “ejectives are favored at high elevations because they are easier to articulate in such locales [due to lower external air pressure], and because they attenuate … the rates of water vapor loss in exhaled breath.”

We comment further on these suggestions below.

### 1.5. Resulting motivation for data collection

The studies cited provide the basis for the selection of both linguistic and climatic/environmental variables to include in our analysis. On the linguistic side, we have focused on overall consonant and vowel inventories, as well as some sub-categories, such as the number of obstruents, the presence and number of ejectives and other laryngealized consonants, of velar nasals, and nasalized vowels and vowel length. These linguistic variables have been implicated in proposals relating linguistic to climatic/environmental variables or are known to have biased geographic patterns of distribution that may therefore potentially be linked to local conditions. Since no theoretical reasons have been proposed to expect environmental factors to have influence on the distribution of some of the variables at the end of this list, they may provide a check on the likelihood of adventitious correlations between linguistic and non-linguistic properties. Note that in each case the data on the linguistic side of the equation refers to somewhat abstract categorical values, for example phonemic consonants or vowels and their traits, or contrastive tone levels or contours, and not to the infinite variation that is found in natural speech.

On the climatic/environmental side we have focused on seeking the most reliable data obtainable on temperature, humidity, precipitation, ground cover/vegetation, biomass, and altitude. This involves negotiating issues of what data is available, in what form, for what areas, and over what time spans.

## 2. Materials and equipment

### 2.1. Language data

As noted above, the linguistic data in our database covers the overall size of consonant and vowel inventories and several specific aspects, such as the inclusion of ejective consonants, velar nasals, or front rounded vowels. It also includes information on whether the language is tonal or not and, if tonal, how elaborate the system of tone contrasts is. The complexity of syllable structure is represented by indexes reflecting the maximal elaboration of onsets and codas permitted. Various indices reflecting the overall balance of the language between greater use of vowels or of consonants are also included. These include the vowel index calculated by Everett (2017) for the languages in common in our samples and indexes of ‘consonant heaviness’ reflecting both the number of consonant contrasts and their deployment in simpler or more elaborate strings in syllable onsets and codas.

None of these data are straightforward, as analyses are rarely consensual. Readers are referred to the LAPSyD database (Maddieson et al., 2013, 2013–2023) for some discussion of the choices made in determining the values selected for any given language. Some of the issues concerned are also reviewed in Maddieson (2023). Our linguistic data, as in LAPSyD, represents a single ‘snapshot’ of each language as spoken at a particular place and time. Unlike PHOIBLE, another phonological database (Moran and McCloy, 2019) which includes conflicting analyses of a given language, a single analysis is reached, which may not correspond exactly to any of the published descriptions. The aim is to establish a consistent style of interpretation that minimizes the influence of different theoretical stances in the manner of Dixon’s Basic Linguistic Theory (Dixon, 2009).

### 2.2. Selected environmental and supporting data sources

In addition to providing environmental data relevant to the linguistic hypotheses outlined above, the environmental data sources used in the analysis have been selected based upon the following criteria:

- Global coverage
- Spatial resolution that provides the opportunity to characterize both central tendency (mean and median) and variance (variance, standard deviation, inter-quartile range, percentiles) for an environmental variable within a variably sized catchment surrounding each language
- Temporal coverage that reduces the impact of accelerated change in global climate variables during the late 20th and early 21st centuries while maximizing the availability of data in proximity to the languages included in the analysis.

In preparation for analyzing the relationships between environmental parameters and language characteristics the language attribute data file; five environmental data sources providing eight environmental parameters; and three supporting data sources providing global imagery, terrestrial boundaries, and global temperature anomaly data were used. The resulting set of project data parameters and descriptive information are summarized in Table 1 (language parameters), Table 3 (environmental parameters), and Table 4 (supporting data) and described in greater detail above (language parameters) and in the following sections.

**Table 3:** Environmental parameters.

Environmental parameter (type)	Date (range)	Spatial resolution	Source coordinate reference system	Citation
Annual temperature minimum – °C (point)	1763-Present	n/a	GCS_WGS_84	National Centers for Environmental Information (NCEI) (2020)
Temperature mean – °C (point)	1763-Present	n/a	GCS_WGS_84	National Centers for Environmental Information (NCEI) (2020)
Temperature maximum °C (point)	1763-Present	n/a	GCS_WGS_84	National Centers for Environmental Information (NCEI) (2020)
Precipitation mm (point)	1781-Present	n/a	GCS_WGS_84	National Centers for Environmental Information (NCEI) (2020)
Specific humidity unitless (raster)	1960-Present (monthly)	1.875° E-W 1.88881° -1.90474° N-S	GCS_WGS_84	Kalnay et al. (1996) and NOAA NCEP (2022)
Land cover categorical (raster)	2000	0.008929° 16353 × 40320	GCS_WGS_84	Millennium Ecosystem Assessment (2005)
Above-ground live woody biomass density MegaG/Ha (raster)	2000	0.00025°		Global Forest Watch (2022)
Elevation m (raster)	1994–2005	0.008333° (30-arc second) 288 15°x15° tiles 1800 × 1800/tile	World_Equidistant_Cylindrical	Berry et al. (2010, 2019)

**Table 4:** Supporting data.

Supporting data parameter	Date (range)	Spatial resolution	Source coordinate reference system	Citation
Global satellite imagery mosaic	2004	500 m/pixel at equator	GCS_WGS_84	NASA Earth Observatory (2005)
Global country boundaries	2017	n/a	GCS_WGS_84	Minnesota Population Center (2013)

#### 2.2.1. Environmental parameters

**Figure 3:**
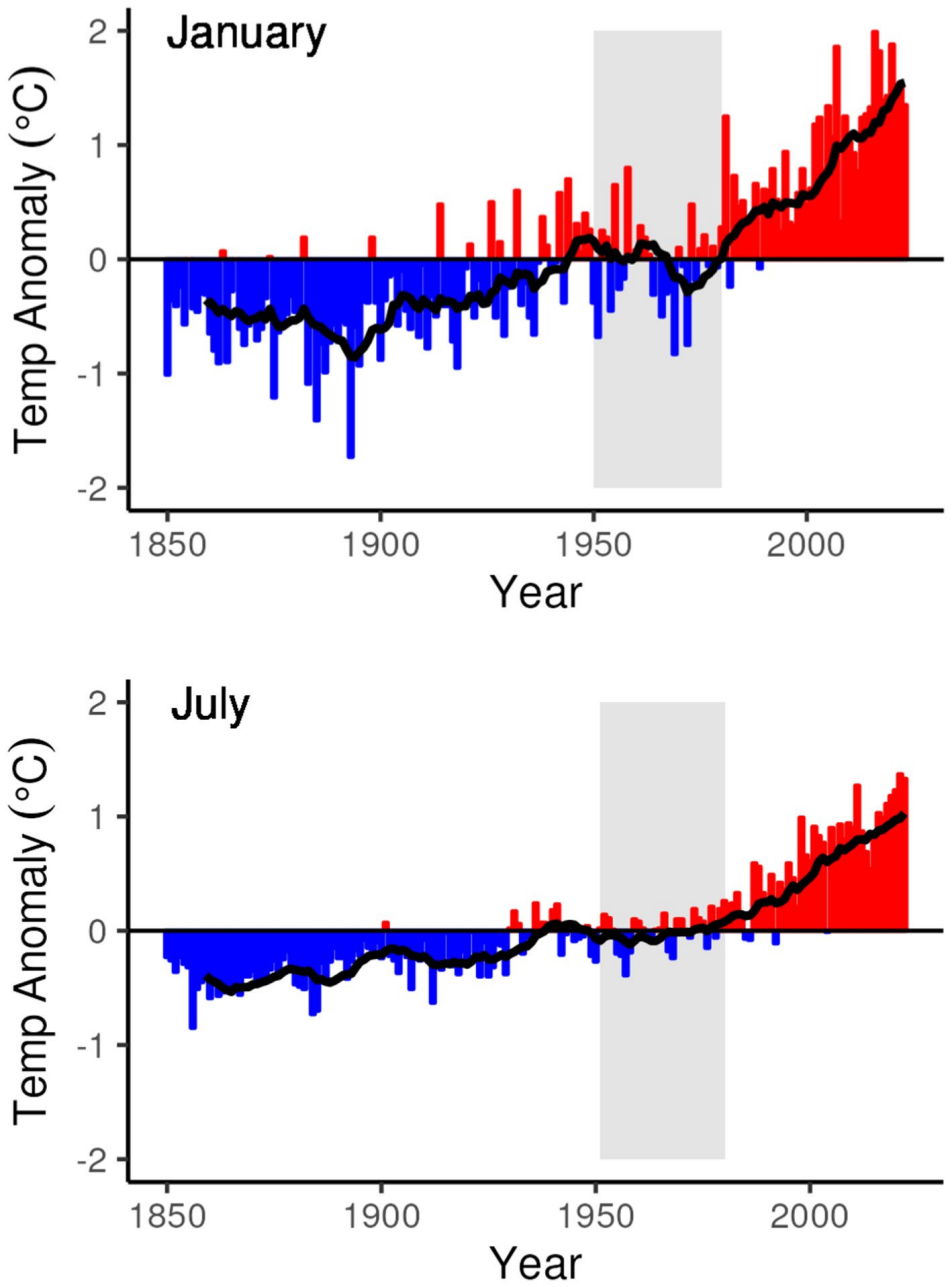
Global land temperature anomaly (from the 1901 to 2000 average) for the months of January and July from 1850 to 2023. Superimposed on the graphs is a 10-year moving average line that smooths out the annual variations for a trailing 10-year period. The 30-year period from 1951 to 1980 for which global weather station data are used for the analytic dataset is highlighted in gray. Data from NCEI Global Time Series, Climate (National Centers for Environmental Information, 2023).

The selection of specific environmental data sources that meet the coverage and resolution requirements outlined above was an exercise in balancing data availability, reduction in bias introduced by global climate change in the 20th and 21st centuries, and anthropogenic land cover change. The trend in global land temperature change, which has increased 0.66°C more than global combined land and ocean temperature (Intergovernmental Panel on Climate Change, 2022, p. 84), is illustrated in Figure 3. The temperature trends illustrated show a gradual increase in temperature until roughly 1980, after which there is a substantial increase in the rate of global temperature increase. The period from 1951 to 1980 represents a period of relatively steady (July) or slightly declining (January) temperatures that approximate the long-term 1901–2000 global average, and as a 30-year period ending in a “tens” year allows for comparison and alignment with other “climate normal” values calculated following the World Meteorological Organization standard (World Meteorological Organization, 1989).

**Figure 4:**
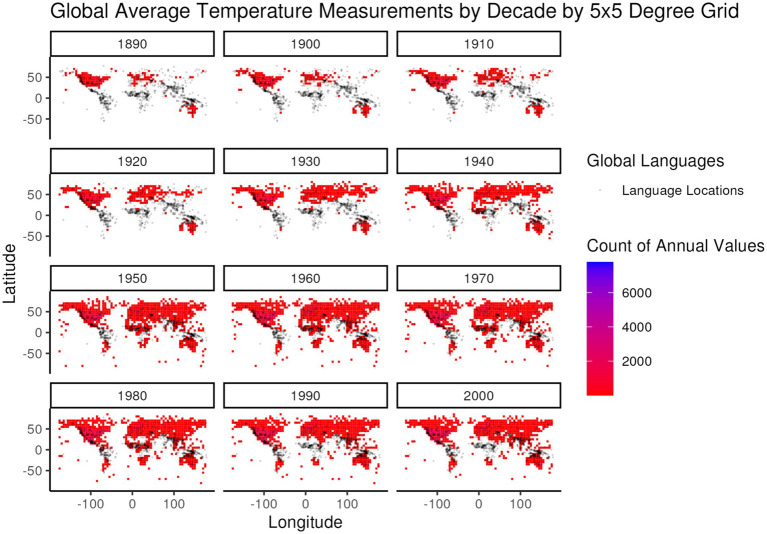
Global frequency of annual average temperature values from the Global Summary of the Year (Lawrimore et al., 2016) dataset, summed over global 5 × 5 degree regions, by decade of measurement (e.g., all measurements from 1950 to 1959 are included in the 1950 decade). The color gradient of frequencies is overlaid by the distribution of languages (gray dots) in the developed global dataset for comparison of language locations to the distribution of temperature measurements.

**Figure 5:**
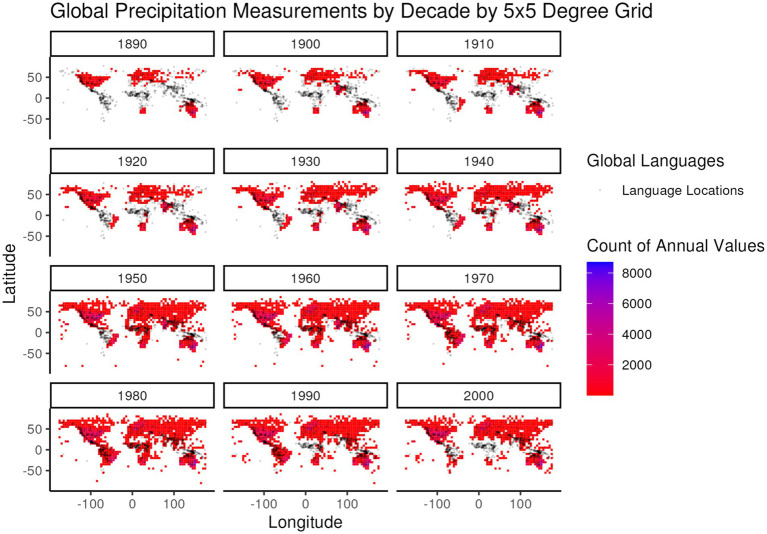
Global frequency of annual precipitation values from the Global Summary of the Year (Lawrimore et al., 2016) dataset, summed over global 5 × 5 degree regions, by decade of measurement (e.g., all measurements from 1950 to 1959 are included in the 1950 decade). The color gradient of frequencies is overlaid by the distribution of languages (gray dots) in the developed global dataset for comparison of language locations to the distribution of precipitation measurements.

While 1951–1980 represents a period of relatively steady global temperature and precedes the period from 1980 to present in which the rate of increase for global temperature accelerated, it still represents a period of higher global temperatures than earlier in the global instrument record. In selecting this particular 30-year period an additional criterion was considered – global coverage of high-quality weather stations. Figures 4, 5 illustrate the global distribution of temperature and precipitation measurements, respectively, from weather stations that meet the long-term quality requirements of the Global Historical Climatology Network (Menne et al., 2012) that are then summarized in the Global Summary of the Year (Lawrimore et al., 2016) dataset that is used in this analysis. The distribution patterns for both temperature and precipitation measurements show a strong bias towards the global north through the 1940s, with large regions of the global south only starting to fill in during and after the 1950s. Even during the 1950s and beyond the distribution of temperature and precipitation measurements is not the same, as can be seen in the different distributions of temperature and precipitation values in the 1970s in South America and Central Africa.

Based upon the combination of these temporal trend and spatial coverage criteria it was ultimately decided that the period from 1951 to 1980 would best serve the objective of obtaining comparable instrumental temperature and precipitation data for the largest number of global language locations while reducing the impacts of global climate change. Unfortunately, this well-motivated choice limits the number of temperature data points available for further processing.

The final number of weather stations used in the calculation of estimated temperature and precipitation values for each language location is dependent upon the specific shape of the sampling region around each language. All other environmental parameters are likewise summarized for each language’s sampling region. The method for calculating the language’s sampling region (i.e., the range-and coastline-truncated Voronoi cell for each language) is outlined below. Summary data for the individual environmental parameters, including the number of languages for which that parameter is calculated, are also provided in that section.

The same temporal selection criteria were used in the extraction of monthly specific humidity data (expressed as a unitless ratio of the weight of water vapor within a given weight of air) from the National Oceanic and Atmospheric Administration (NOAA) global Climate Data Assimilation System (CDAS) “above ground qa” dataset (Kalnay et al., 1996; NOAA NCEP, 2022). As this dataset includes gridded monthly values from January 1960 through present, only the subset from 1960 through 1980 was included in this analysis as specific humidity increases with increasing temperature when an air mass is at equilibrium with a source of water vapor, and comparability with the used instrumental weather data was desired.

The land cover data used in the current analytic system were generated as part of the Millennium Ecosystem Assessment (2005) and includes a globally harmonized land cover classification system that includes “coastal, cultivated, forest and woodlands, inland water bodies, islands, marine, mountains (elevation), polar, and urban” categories. This global dataset represents the distribution of these land cover classes in roughly the year 2000 and as a result reflects historic changes in landcover that have occurred due to natural and human-caused sources. Examples of the potential historic changes (from 1765 to 2000) include significant reductions in primary forest (45.4 million km2 [Mkm] to 20.8–22.5 Mkm), increases in secondary forest area (0.0 Mkm to 7.0–7.9 Mkm), significant increases in cropland (3.5 Mkm to 5.0–32.1 Mkm), moderate increases in pastureland (4.2 Mkm to 5–6.9 Mkm), and relatively smaller changes in savanna, shrubland, and other land cover classes. While there was a significant increase in urban land cover since 1765 (0.0 Mkm to <0.1–0.5 Mkm), the scale of urban land change is minor when compared to other land cover classes (Meiyappan and Jain, 2012; Table 4). While the Historical Land-Cover Change and Land-Use Conversions Global Dataset distributed by NOAA’s National Centers for Environmental Information (National Centers for Environmental Information, 2012) provides a global 0.5 × 0.5-degree gridded dataset for the estimated land cover data from 1770 to 2010, the uncertainty and limitations cited by Meiyappan and Jain (2012, pp. 133–134) in the modelled land cover data complicate their use in this analysis.

The Above Ground Live Woody Biomass Density (AGB) dataset was created by and continues to be maintained by Global Forest Watch (Global Forest Watch, 2022) and represents, in the case of the version used in this project, the megagrams of AGB per hectare on a global scale at an approximately 30-m (~1 arc-second, or 0.00025 degree at the equator) spatial resolution for the year 2000. The source dataset consists of 280 separate files that must be combined prior to their use analytically. Two lower spatial resolution datasets are available through the Oak Ridge National Laboratory’s Distributed Active Archive for Biogeochemical Dynamics biomass data collection (Oak Ridge National Laboratory, Distributed Active Archive Center for Biogeochemical Dynamics, 2023). The first represents biomass (among other parameters) at monthly and yearly time steps between 1900 and 2010 at a global 0.5-degree spatial resolution (Huntzinger et al., 2018). The second provides forest biomass (and other parameters) at 5-year intervals between 1950 and 2015 at a near-global (70-degrees S to 70-degrees N, 180-degrees W, 180-degrees E) scale at a 1 × 1 degree spatial resolution (Hengeveld et al., 2015). While these alternative datasets may provide some mitigation to global land-cover and associated biomass change, they would do so at the expense of the higher spatial resolution provided by the currently used AGB dataset. In the long run these alternative datasets may be useful, but assessment of their utility remains for future analysis.

The Altimeter Corrected Elevations, Version 2 (ACE2) global elevation model (Berry et al., 2010, 2019) is used in this analysis system for modeling the elevation within the sampling region for each language in the analysis. This dataset is derived from multiple remote sensing and ground observation data sources to provide global coverage at multiple spatial resolutions ranging from 3, 9 and 30 arc-seconds, to 5 arc-minutes. The 30 arc-second version of the dataset was selected for this analytic system as it provides relatively high spatial resolution (~1 × 1 km at the equator) while not requiring the substantially higher storage and computational resources that the 3 and 9 arc-second data would. While the data from which this dataset is derived were collected between 1995 and 2005, the overall combined elevation model is not as sensitive to the historic trends introduced by global climate change and is assumed to provide a reasonable representation of the terrain within which the languages in the system developed.

While each of these datasets provide the required source materials for performing analyses of the relationship between language characteristics and the environments within which they developed, each requires additional processing to allow for integration with the language data developed for the project. The following sections discuss the data management and analytic strategy developed for the project and describes the processing steps and resulting derived data products that allow for language-environment relationship hypothesis testing.

### 2.3. Computational tools

To minimize the barriers to potential reuse of the data and computational methods developed for this project a number of Open Source (The Open Source Initiative, 2006) software tools were used. The tools used play multiple roles in the overall system: defining the analytic environment itself in a way that allows automated deployment of the full toolkit on a new system; scripting tools that support the development of reproducible/re-executable command sequences that allow for efficient iterative development and reproduction of results; and specialized analytic tools that support the specific data processing, analysis, and visualization needs of geospatial data.

The portability and capability for deploying the full analytic framework developed for this project onto new systems is enabled through the use of the Open-Source Docker platform (Docker Incorporated, 2021; Docker Incorporated, 2022) and its use of custom “Dockerfile” documents that define how the analytic environment should be created within a “container” that provides a self-contained execution environment that can be run on a wide variety of computer systems. All of the code and configuration files are included in a public GitHub repository (Benedict, 2022a) that is preserved and citable through the Zenodo repository (Benedict and Maddieson, 2022a). This method of encapsulation allows for flexible deployment into new computational environments when needed. This capability has been demonstrated over the course of the development of the system through its use on desktop and laptop Macintosh computers and most recently on a Linux server hosted in Digital Ocean’s cloud environment (Digital Ocean, LLC, 2023).

The Open-Source R programming language (The R Foundation, 2023) and the associated RStudio integrated development environment (Posit, 2023) has been used as the primary scripting and analytic environment for this project as it provides a fully functional programming environment for solving a wide array of analytic and data management challenges while also having specific tools for integrating with the GRASS geographic information system (GRASS Development Team, 2023a) analytic tools selected for the project.

GRASS GIS was selected as the primary geospatial data management and processing environment as it provides a comprehensive set of geoprocessing functions that are designed to be executed in a lightweight environment within which a small set of core environmental variables can be defined (i.e., the location of executable files, the location within the data storage system where data are stored, the current coordinate reference system, etc.) and within which individual GRASS commands can be executed. This enables the integration of GRASS geoprocessing functionality into external tools such as R scripts (as done in this project), Python or Linux shell scripting tools, or other desktop GIS applications such as QGIS (QGIS Project, 2023).

All of these computational tools are automatically configured and installed through the configuration files, setup scripts, and analytic scripts that are maintained and shared through both the GitHub repository (Benedict, 2022a) for ongoing development and the Zenodo archive for preservation and citation (Benedict and Maddieson, 2022a). For convenience, the “raw” data files downloaded from the diverse data sources cited in Tables 3, 4 used to initialize the analytic environment are stored in a publicly accessible object storage system in Digital Ocean’s cloud, but those source files can also be downloaded directly from the providers of those data and placed wherever needed by a researcher desiring to run the system. The language data used in this system are also managed in a public GitHub repository (Maddieson and Benedict, 2022b) for ongoing development and preserved and made citable through snapshots in the Zenodo repository (Maddieson and Benedict, 2022a).

This combination of automated system configuration, public access analytic code and source data, and Open-Source technologies for the execution environment enables maximum opportunity for adaptation and reuse of the developed system and its components, both for the current project, but also for future analytic work.

## 3. Methods

### 3.1. Data management and analytic strategy

In support of addressing the linguistic questions outlined above a methodological approach has been adopted that:

- Maximizes efficient re-execution of data ingest and analytic code during the project’s iteration on the source data and analytic approach
- Employs a hybrid analytic tool set that combines multiple Open-Source tools into an analytic environment that supports automation and encapsulation of both data and analytic code. Information about the specific tools and their roles is provided in the Computational Tools section above
- Uses analytic code that allows for selective re-execution of analysis steps, enabling accelerated code revision and re-execution cycles during development.

During the development of the system described here the source language database was under continuous development and needs for performing both quality control and preliminary analyses were continuously evolving. To meet this need an organizational structure for analytic raw material (i.e., data obtained from source data providers), scripts defining the data processing, management, and analytic steps, and derived products (i.e., generated output and derived data products) was established. This structure is automatically created as part of the automated analytic environment creation process that is defined in the “Dockerfile” and executed by the “build.sh” shell script, both of which are in the top level of the project GitHub repository. Execution of these setup and configuration files creates a high-level directory structure that includes folders for: raw data, scripts, output data and images, the GRASS GIS data store, and a temporary directory for content that can be reused as needed. This structure allows for a strict separation between source data and analytic processes and products, ensuring that the data from which the analyses are derived are unmodified and can be reused to initialize updated analyses.

Given the iterative development process employed in the development of the language dataset and analytic code, the R scripts developed for the project are separated into sets that address different needs:

- Reusable code that is included in multiple scripts to provide a common operational environment for multiple analytic processes. These scripts have a “00_” prefix in the scripts folder in the generated analytic environment
- Setup scripts that usually only need to be run once within the analytic environment to perform additional setup steps. These scripts have a “01_” prefix
- Data import scripts that can be run, and rerun as needed, to import source data into the analytic environment for further analysis and visualization. These scripts are separately run for each source dataset allowing for targeted re-ingest of source data if/when needed. These scripts have a “02_” prefix
- General purpose data visualization scripts that generate output visualizations of source data for use in both quality assessment/quality control (QA/QC) and basic interpretation of data. These scripts have a “03_” prefix
- Data extraction scripts that can be run and rerun as needed when any of the data being extracted change and to extract data from multiple processed data sources into a combined dataset that can be used analytically. In the current analytic environment, there is a single data extraction script that generates summary statistics for multiple environmental variables and generates an output comma separated value (CSV) file that combines these environmental variables with the language variables for each language in our analytic set. These scripts have a” 04_” prefix
- Data analysis and visualization scripts that perform more specialized analytic processes that are customized to meet more targeted QA/QC, data analysis, and data visualization needs – typically for specific subsets of data for which more specialized analytic methods are appropriate. These scripts have a “05_” prefix.

Descriptions of the different scripts and their actions are provided in the README.md file in the shared GitHub repository (Benedict, 2022b). The separation of the developed data management, processing, and analysis code allows for granular execution and re-execution of specific processing workflows without incurring the cost of re-running the complete set of processes from beginning to end. This has resulted in a highly efficient development and execution environment in which only the analysis steps required by a targeted data change or updated analytic process need to be run, often resulting in hours of saved execution time when compared to the alternative of running the full set of scripts.

### 3.2. Data processing and analysis

The data processing and analysis performed in the development of the current analytic environment includes two high-level processes: the import and processing of the language dataset to allow for extraction of environmental parameters for each language in the dataset, and the import and processing of the source environmental datasets to enable the extraction of statistical summaries of those environmental parameters for integration with the language parameters for further analysis.

**Figure 6:**
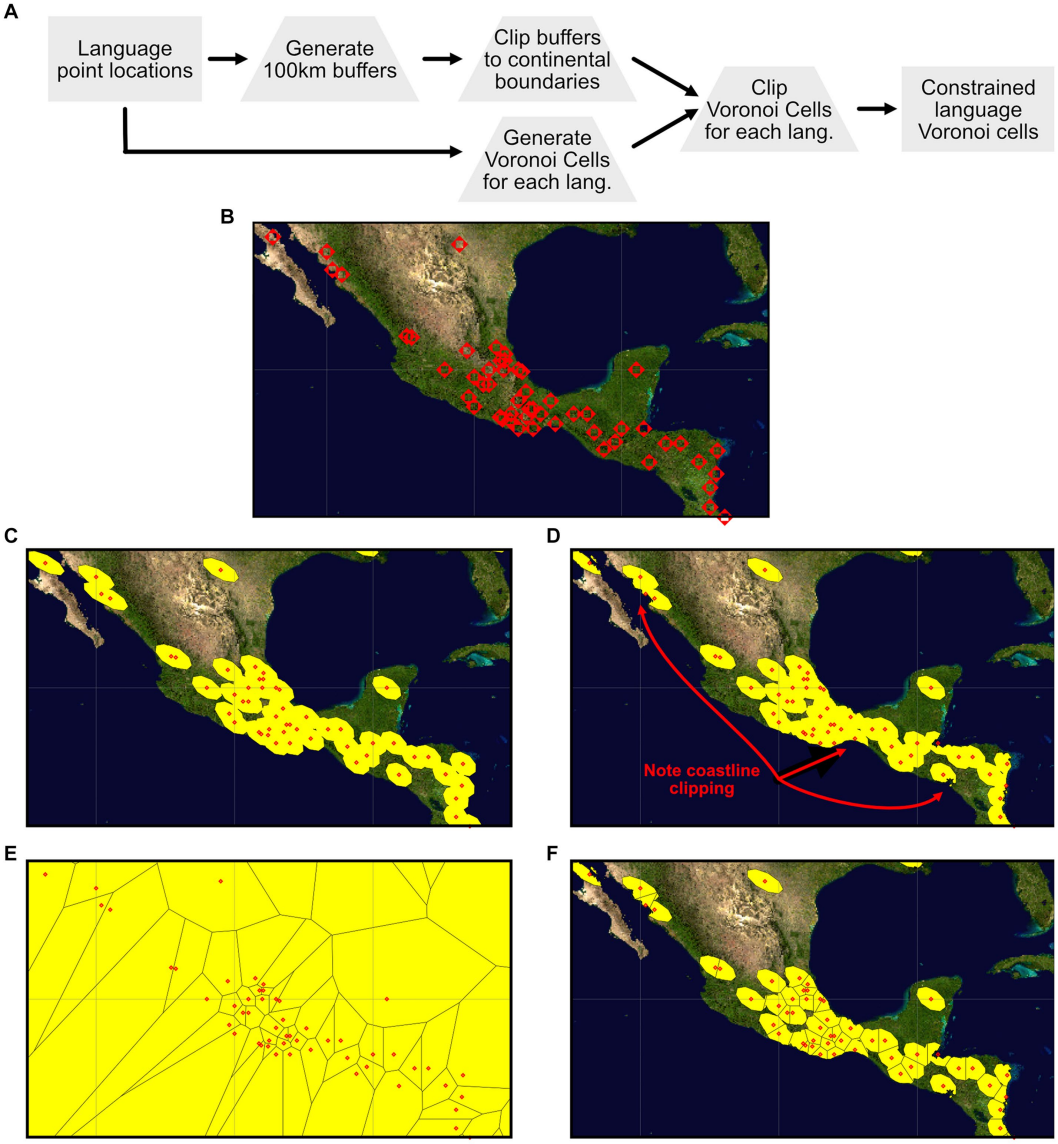
Workflow for generating constrained Voronoi cells for each language in the dataset. **(A)** Illustrates the overall conceptual workflow, starting with the latitude-longitude point locations for each language, 100 km buffers and Voronoi polygons around each language point, clipping the 100 km buffers to the coastlines to exclude off-shore areas, clipping the Voronoi diagram to the clipped 100 km buffer regions, ultimately producing the final constrained Voronoi cells for environmental data extraction. **(B)** Through **(F)** illustrate a region of Central America showing each stage of this process: **(B)** Language point locations within the Central America sub-region, **(C)** the 100 km buffers surrounding each language, **(D)** the 100 km buffers clipped to eliminate off-shore areas, **(E)** the based Voronoi diagram for the points in the Central America sub-region, and **(F)** the final Voronoi cell areas that have been clipped to the areas of the clipped 100 km buffer regions.

The source data for the languages in the dataset include point latitude and longitude values for each language. Our objective in extracting environmental parameters for each language was to develop an understanding of the environment surrounding each language point while also, to the extent possible, maintaining independence of the environmental parameters extracted for each language. This was accomplished through the development of what we are referring to as “constrained Voronoi cells” for each language, with the combined collection of cells collectively referred to as a Voronoi diagram (Atsuyuki et al., 2000). The developed Voronoi diagram is conceptually similar to the bounded Voronoi diagrams described by Tournois et al. (2010), but due to the specific implementation of the GRASS GIS Voronoi diagram generation function (*v.voronoi*) (GRASS Development Team, 2023b), which lacks the ability to specify a more complex bounding geometry than a simple rectangular bounding box, the Voronoi cells used for the language environmental parameter extraction are produced by a “simple” intersection of a global rectangular Voronoi diagram with a previously defined constraint GIS layer defined through a combination of 100 km buffers around each language location and coastlines extracted from the IPUMS International global world map national boundaries dataset (Minnesota Population Center, 2013). The 100 km buffer size around each language was selected to provide a reasonable sampling region around each language point while still focusing the extraction of environmental data to a relatively local region around each language. In coastal zones and areas of high language density the sampling region defined by this 100 km buffer is further reduced in size based on the exclusion of offshore areas and the partitioning of space by the Voronoi diagram generation process. The overall process of developing the final set of sample regions for the language collection is illustrated in Figure 6.

Inspection of the data extraction regions generated by the process illustrated in Figure 6 highlights some artifacts of the process that have a small impact on the environmental values extracted for each language. First, as can be seen if one closely examines some of the final Voronoi cells in Figure 6F, the partitioning of the sample area for each language is first defined by the Voronoi cell boundary, and then by the *combined* 100 km buffer areas. This has the effect of slightly extending the final sampling area for some languages beyond that language’s 100 km buffer as an artifact of the specific structure of the spatial relationships between each language, its adjacent languages, and the shape of adjacent constraint boundaries. This issue yields 17 languages (1.7% of the sample of 1,003 languages) that have a sample area greater than the base 100 km buffer area, with those 17 languages ranging from 1.14 to 2.75 times the base area. The detailed understanding of the circumstances for these inflated sample areas remains under development. An additional artifact that is visible in Figures 6C,D,F is the elongation and angle of the sample areas. These are a product of the process of developing the 100 km buffers in the World Sinusoidal (MapTiler, 2023a) coordinate reference system that is optimized to maintain area across a wide range of latitudes and longitudes at the expense of shape and direction. When transformed back into the geographic coordinate reference system (MapTiler, 2023b) these equal-area sampling regions end up reflecting shape and orientation distortion that is a byproduct of the differences between these different coordinate reference systems.

The source environmental data (Table 3 summarized the characteristics of these data) originate as either point data (weather stations for which there are annual meteorological summary data) or continuous data represented as raster data that are provided as one or more data tiles (elevation, land cover, biomass, and specific humidity). The summarization methods are used for each category of data are as follows:

- Point data are summarized by identifying the station locations that are located within the sampling region for each language and calculating the mean and sample size (i.e., the number of annual values included in the calculation) for each parameter of interest (minimum annual temperature, maximum annual temperature, average annual temperature all in degrees C; and annual accumulated precipitation in mm)
- Raster data are summarized by calculating summary statistics for the raster cells that fall within the sample region for each language. The types of statistics calculated depend on the types of data represented by the raster
  - For rasters representing numeric data (i.e., elevation, biomass, and specific humidity) summary statistics include the number of raster cells contributing to the statistic, the number of null cells within the region, measures of dispersion including average and median, and measures of dispersion including minimum, maximum, range, first-and third-quartiles, standard deviation, variance, and coefficient of variation
  - For rasters representing qualitative data (i.e., land cover classes) the number of cells representing each land cover class are counted and included in the output dataset as a separate data column representing the number of cells of that type within the language sample region.

The dataset that is generated as a result of these calculations is internally stored in the analytic system as a polygon GIS data layer in which each polygon represents the sampling region for each language and includes all of the language and summary environmental variables as attributes. To enable analysis of the relationships between language and environmental variables the attributes associated with each polygon are exported as a row in a comma-separated-value (CSV) file (Benedict and Maddieson, 2022b).

The generated CSV file contains all of the language variable values described in Table 1 combined with the statistical summaries for the environmental data described in Table 3. All of the environmental variables are prefixed with a “v_” followed by a short-name for the environmental variable being summarized: “elev” for elevation; “biomass” for biomass; “lc_tall,” “lc_med,” “lc_short,” “lc_water,” and “lc_snow” for land cover classes for tall, medium, and short vegetation, water, and snow; “prcp” for precipitation, and “tmin,” “tmax,” and “tavg” for annual average minimum, maximum, and average temperature. The next element in the variable names represents the units of measure for the variable: “m” for meters, “MgHa” for mega-grams/hectare, “ct” for count, and “dC” for degrees C. The final element in the variable names represents the summary statistic/aggregation method: “number” or “ct” for the number of contributing values, “nulls_cells” for the number of cells containing a NULL value, “minimum” for the minimum value, “maximum” for the maximum value, “range” for the range of values, “average” and “avg” for average, “std_dev” for the standard deviation, “variance” for the variance, “coeff_var” for the coefficient of variation, “first_quartile” for the first quartile, “median” for the median, and “third_quartile” for the third quartile. Table 2 presents a sample of the derived environmental variables included in the exported CSV file, demonstrating the specific pattern for the variable names in the output file and the descriptive information for each variable.

In support of the integration of language relatedness into analyses of the relationship between environmental and linguistic attributes, the combined data documented in Table 2 were used to calculate the differences (distance) between selected linguistic and environmental attributes for language pairs for which linguistic relatedness have been defined (see Controlling for Inheritance above). Linguistic and environmental distances for each language pair are calculated using the R ‘ecodist’ package (Goslee and Urban, 2007, 2022; Goslee, 2010) which supports the calculation of similarity distances for single and multiple variables and performing dissimilarity analyses based on those distances, with the calculated variable distances ultimately being merged with the previously defined language pair distance values. Figure 2 illustrates two examples of the resulting distributions of linguistic and environmental difference values for different degrees of language relatedness.

## 4. Results

### 4.1. Correlating environmental and linguistic data

**Figure 7:**
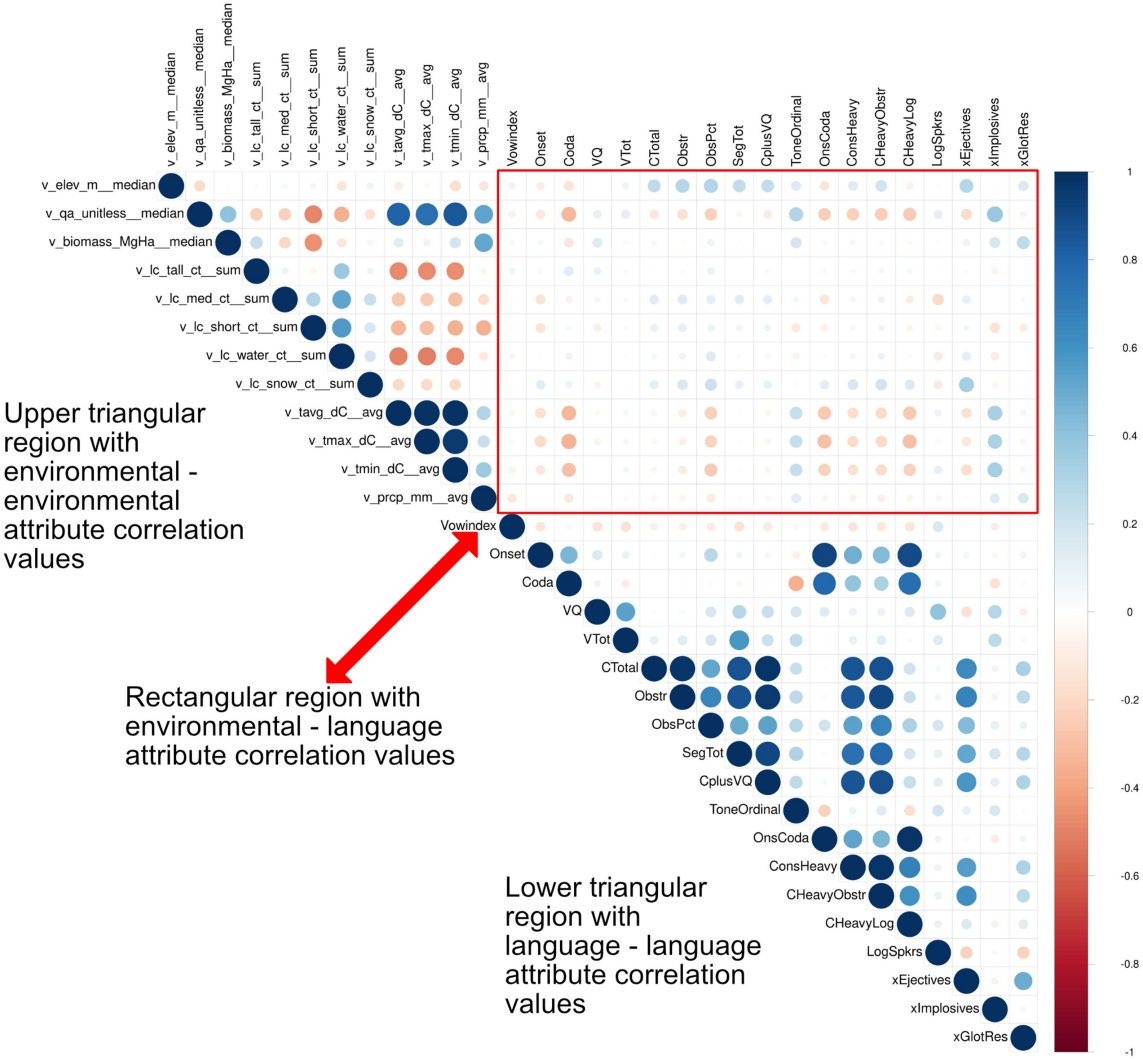
Correlogram illustrating the pairwise Pierson Correlation Coefficients (ranging from −1 to +1 indicating a negative and positive correlation respectively, with a 0-value indicating no relationship between the two variables). The size of the dots and their color saturation in the figure reflect the magnitude of the correlation coefficient (i.e., dots increase in size as the correlation coefficient approaches −1 or +1). The hue of the dots indicates the direction of the correlation, with red hues indicating negative correlation and blue hues indicating positive correlation. Table 1 provides a description of the language attributes and their names. Table 2 provides a description of the environmental variables and their names, units, and associated aggregation methods.

Looking at raw data, as can be seen in Figure 7, there are many evident correlations between the various linguistic and environmental/climatic features as well as among the latter. Some of the relations between linguistic and environmental/climatic variables may be fortuitous rather than principled. In this section we review the specific proposals that were discussed above, before proceeding to discuss additional correlations that might or might not be random.

### 4.2. Replications

**Figure 8:**
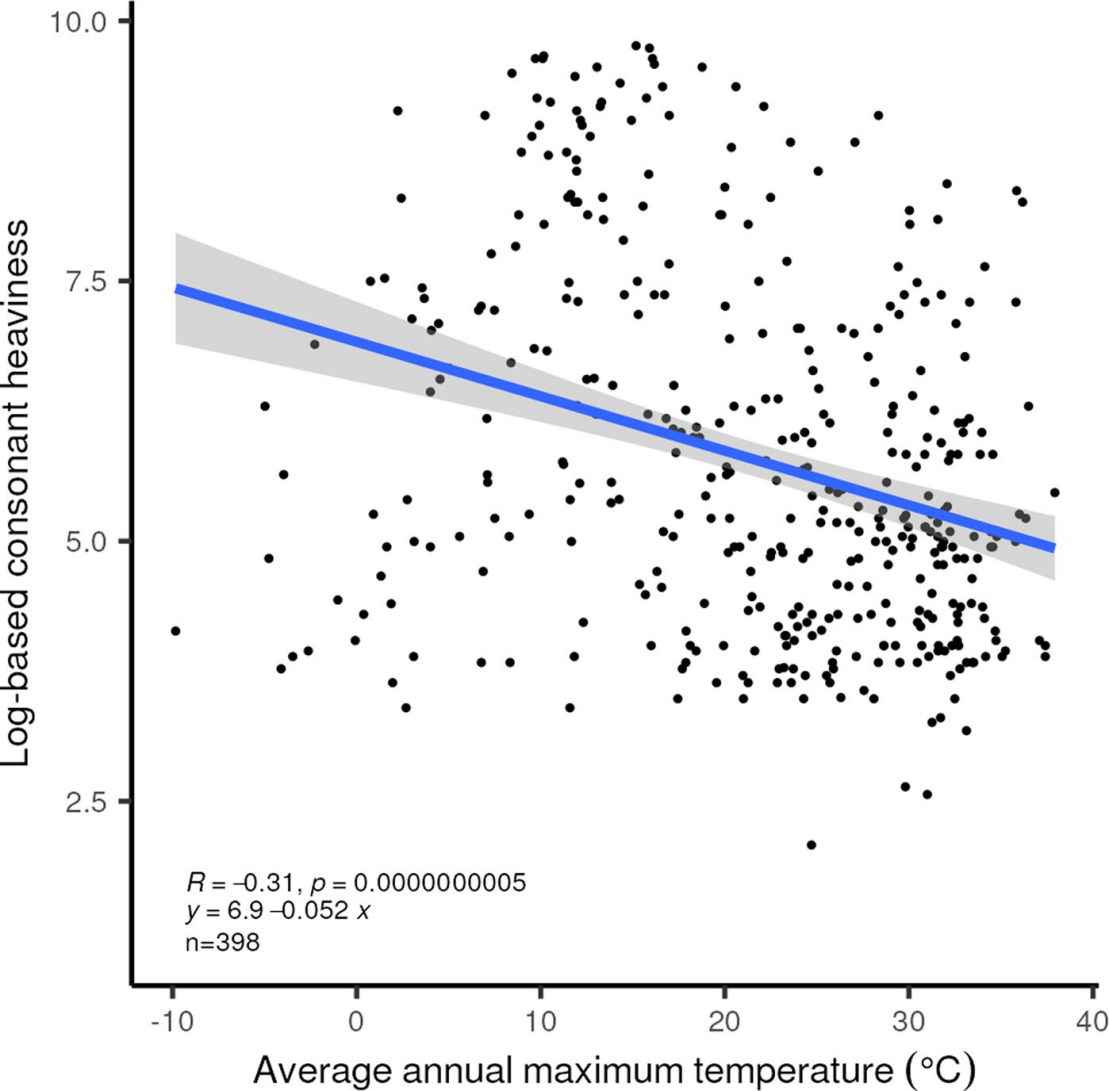
Linear correlation between the log-based consonant heaviness index and average maximum annual temperature for the 398 languages in our set for which ground-based temperature records are available. *R*^2^ = 0.096, *p* < 0.0001.

We have re-checked in a simple fashion the major proposals relating linguistic and non-linguistic variables reviewed in an earlier section, apart from the CV frequency claim in Munroe et al. (1996). Our newly assembled dataset confirms a relationship between smaller overall consonant inventory size plus syllable complexity (“Consonant Heaviness”) and higher maximum temperature in the locality of the language (Figure 8). Lower values of Consonant Heaviness are also associated with higher precipitation and denser biomass, as noted by Maddieson and Coupé (2015).

**Figure 9:**
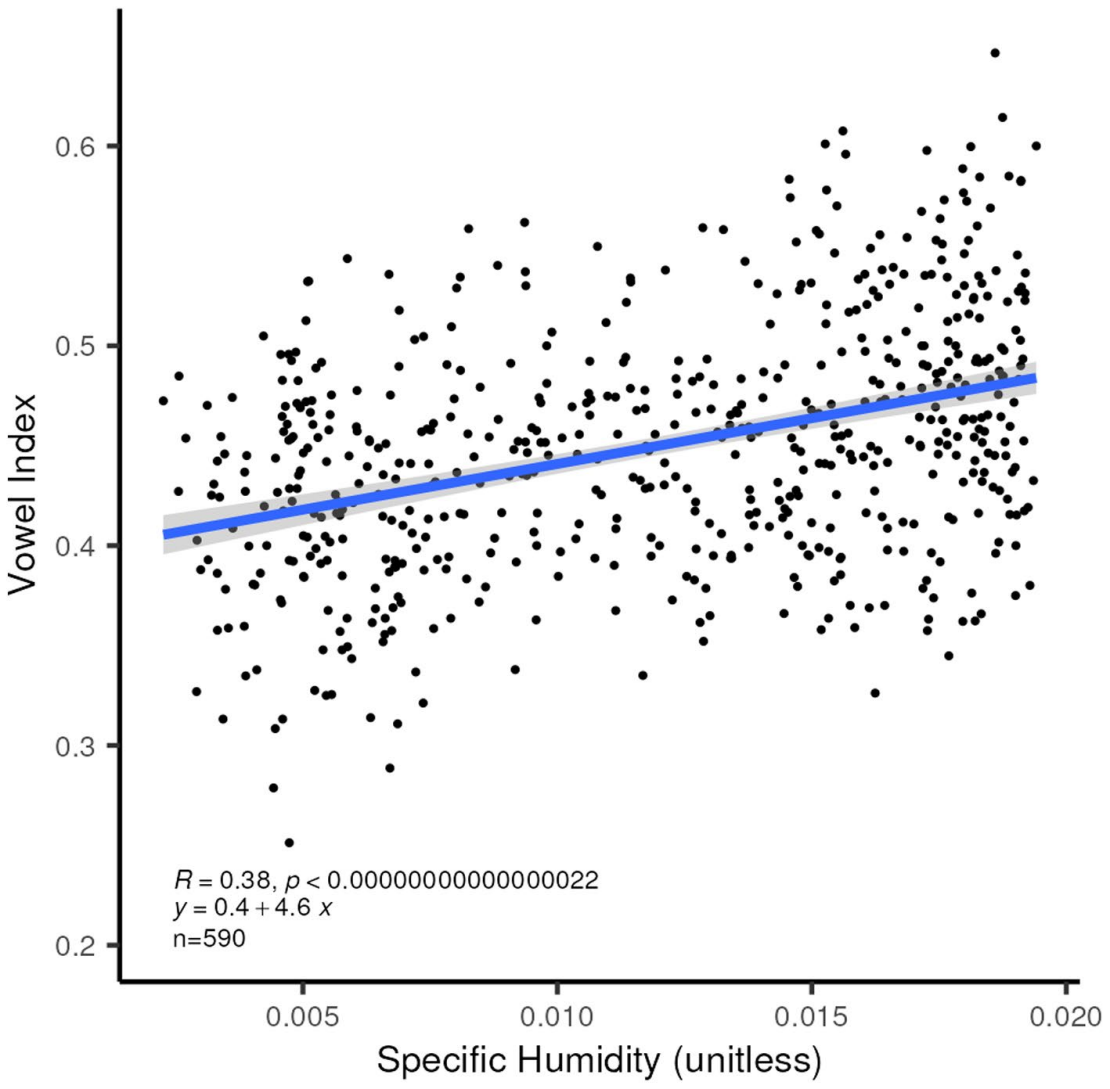
Linear correlation between Vowel Index (Everett, 2017) and mean specific humidity for the 590 languages in our set for which this index is provided. *R*^2^ = 0.144, *p* < 0.0001.

We also confirm the relationship posited by Everett (2017) between higher humidity and greater reliance on vowels in the lexicon (Figure 9). In addition, this index correlates significantly with higher average maximum temperature, as is expected given the fact that the Vowel Index and Consonant Heaviness are measuring related properties of the languages (the *R*^2^ value for the correlation between these two indices is 0.3067), and that temperate and humidity are highly correlated with each other.

**Figure 10:**
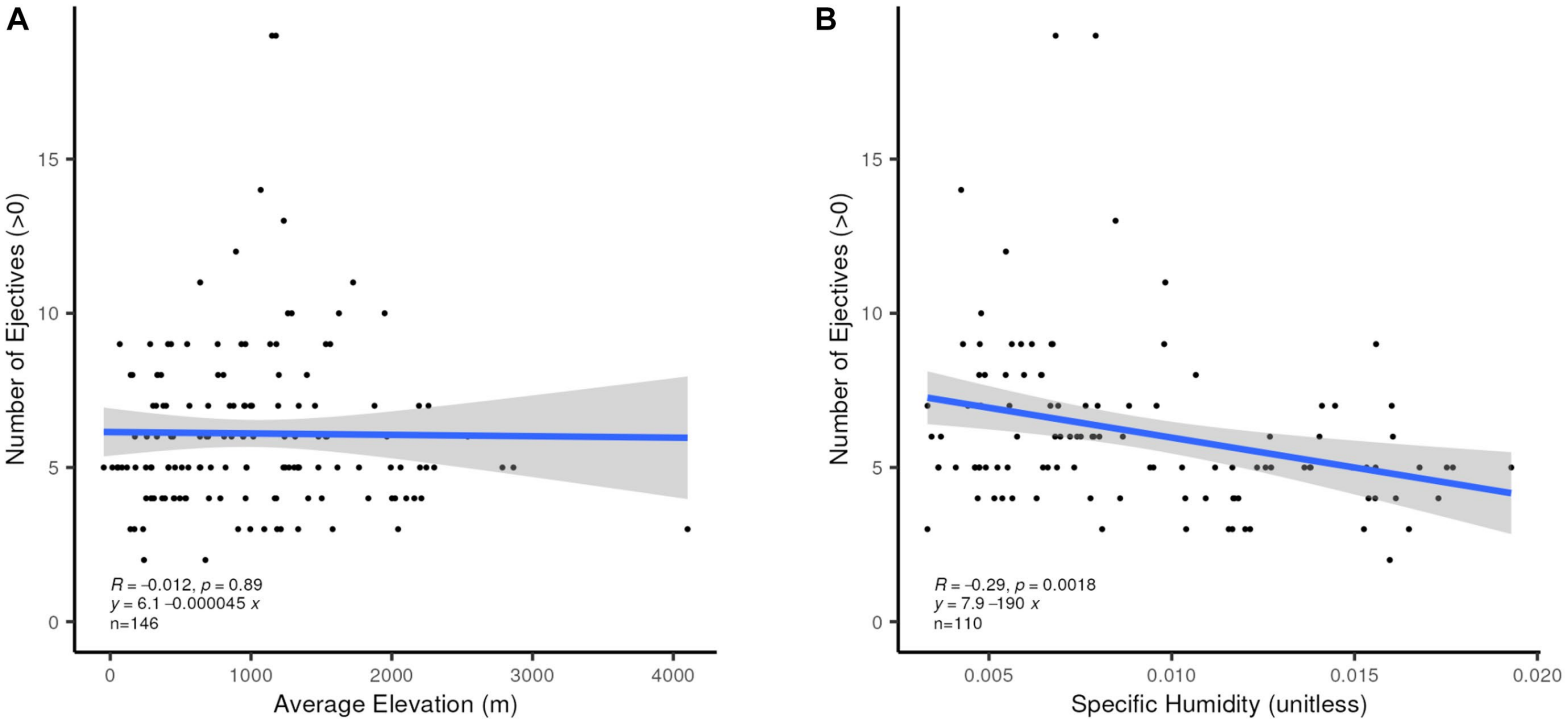
**(A)** Linear correlation between number of ejectives and average altitude (m). **(B)** Linear correlation between number of ejectives and specific humidity (unitless).

We also confirm finding a simple relationship between the presence of ejectives and higher altitude, as proposed in Everett (2013), whether the average or the maximum altitude in the area defined for each language is used. There is, however, a very unbalanced number of languages in the two sets, those with and those without ejectives. This connection has been questioned by Urban and Moran (2021). We posit as a corollary to Everett’s proposal that a larger number of ejectives in the inventory might be expected to occur the higher the altitude at which a language is spoken. This is not confirmed, as Figure 10A shows. When the number of ejectives in those languages which have any (146 languages) is analyzed, there is no relation between increasing altitude and increasing numbers of ejectives, but some correlation is found between fewer ejectives and lower humidity (*R*^2^ = 0.0841, *p* = 0.0018) for the 110 languages which have ejectives and associated specific humidity values, as shown in Figure 10B. As noted before, smaller consonant inventories overall are broadly associated with higher humidity, and ejectives typically occur in larger consonant inventories.

**Figure 11:**
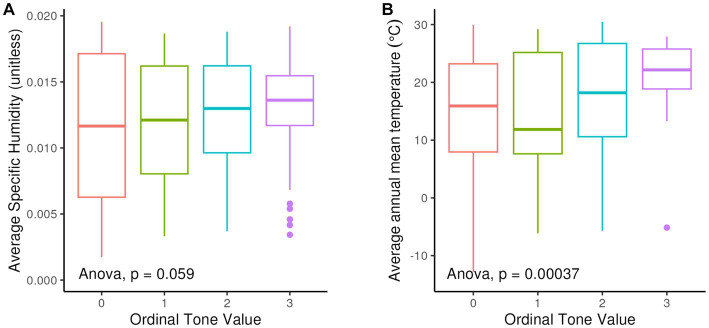
**(A)** Boxplots of average specific humidity by tone system complexity; 0 = non-tonal languages – 3 = complex tone system (more than 3 tones). **(B)** Boxplots of average mean annual temperature by tone system complexity; 0 = nontonal languages – 3 = complex tone system (more than 3 tones).

As for the proposed relationship between tone and higher humidity (Everett et al., 2015, 2016), we find an overall increase in the average humidity values with increasing complexity of tone systems (Figure 11). As shown in Figure 11, there is a sharper divide between non-tonal or simple-tone (Level 1) languages, and those with 3 or more tones (Levels 2 and 3) with respect to average temperature, though this comparison covers fewer languages.

### 4.3. Planned versus unplanned comparisons

For each of the replicated results above an explanatory account has been offered to support the relationships found, and in a statistical sense are considered planned comparisons. Assessing the robustness of the reasoning provided seems to us the best answer to the fourth issue noted in our Introduction: how to distinguish spurious from theoretically grounded correlations. We particularly consider that coherence of results across different traits can provide persuasive support for proposed environmental-linguistic links, as discussed in section 4.4 below. We note that it is not hard to find other correlations (statistical unplanned comparisons) for which no obvious reason is apparent. For example, the occurrence of front rounded vowels is quite strongly associated with lower humidity (χ^2^ significance value *p* = 0.0001). Front rounded vowels tend to occur in larger inventories of basic vowels (mean of 8.63 for the 64 languages in our sample with one or more front rounded vowels vs. 5.85 for the remainder) but there is no overall connection between vowel inventory size and humidity. Hence this is not a special case of a general trend.

The presence of a velar nasal (/ŋ/) in the consonant inventory is associated with lower average biomass (χ^2^ significance value *p* = 0.0016). Velar nasals occur in fewer inventories than bilabial or coronal ones – in about 53%, compared to about 95% in our language sample – so they might well be expected to be more likely to be found where consonant inventories tend to be larger, as there is an overall trend for cross-linguistically rarer consonants to occur in larger inventories (Lindblom and Maddieson, 1988). As noted above, larger consonant inventories tend to occur in areas in which biomass has lower values. However, /ŋ/ is an exception to this overall trend as mean consonant inventory size is slightly smaller if it is present, 22.4, than when it is absent, 23.5. But there is no reason to think that low biomass has any specific influence on the presence of this individual type of consonant.

Somewhat more ambiguously, languages with contrastive vowel length for some or all of their vowel qualities are associated with lower density of vegetation as well as lower humidity. The presence of a set of distinct long vowels would be expected to increase the ‘vowel heaviness’ of a language [although this property does not enter into the Vowel Index calculated in Everett (2013) which ignores vowel length], and it might therefore be expected to occur more frequently in areas that favor lower levels of our Consonant Heaviness variables, that is, *more densely* vegetated locations.

Relationships such as these indicate that it might be the case that some of the environmentally related distributions of linguistic properties that have been discussed in the literature may be spurious correlations or are part of larger linguistic patterns. In particular, it seems probable that more specific properties – the occurrence of ejectives, for example – are less likely to be by themselves influenced by where a language is spoken than to be aspects of more general characteristics, such as the overall balance between consonants and vowels or of simple versus complex phonotactics – properties that are reflected in measures such our Consonant Heaviness Index or, less directly, in Everett’s Vowel Index. We address the reasons for this view in the following section.

### 4.4. Globality of speaker-oriented and listener-oriented perspectives

Speech communication ordinarily involves an interaction between speaker and listener. A common view is that there is a trade-off between economy of effort and the need to maintain distinctiveness in this interaction (e.g., Martinet, 1955; Lindblom, 1990). Both requirements are seen as constraints on the speaker, who wants to minimize effort but not so far that the message becomes unclear to a listener. As Everett and others have suggested, it is quite possible that there are other factors affecting the speaker that may not be linked to either effort or communicative effectiveness, but instead to ambient conditions. In addition, as Ohala (1981, 2012) has notably pointed out, the listener has an important but to some extent passive role. A listener hears incoming speech but both inherent properties of the signal and the conditions surrounding the transmission lead to imperfect retrieval of all the characteristics of the utterance, and, over time, these misperceptions may contribute to changes in what is taken to be the target pronunciation. However, when ambient conditions are posited as affecting either the production or the perception of speech, these must apply to the entirety of the language. So, for example, if there is a sense among people living at higher altitudes that they need to be careful “to mitigate rates of water vapor loss through exhaled air,” one of two possible explanations offered by Everett (2013) for the association of ejectives with higher altitude, then this would be expected to apply across the board. Languages spoken in such areas would therefore also tend to avoid use of aspirated stops and other segments with high airflow requirements, such as trills. In fact, this is not obviously the case: the languages in our sample with aspirated stops are more likely to be found in areas of higher altitude (mean of average altitudes with aspirates 1,142 m, and without 523 m, *p* = 0.0001). If, as argued by Maddieson and Coupé (2015) high temperature and denser vegetation disrupt the coherence of a signal, and degrade higher frequencies in particular, then any aspect of a spoken signal that relies on more precise timing or on distinctions among high-frequency components is at risk. If conservation of water vapor in the body is important at high elevations, then all types of sounds that are expensive in air flow should be disfavored. Rarity or commonness of particular types of sounds due to environmental effects are thus likely to be aspects of a more general overall design, not singular patterns.

## 5. Discussion

The work reported here serves to establish an environment for ongoing research into relationships between climatic and environmental factors as they may impact language design. We have described strategies and problems associated with assembling the data on both sides of the equation and begun to establish a basis for more extensive future work examining these relationships. The products include a framework for processing environmental datasets and aligning them with the linguistic variables. We have established, but not yet applied, a method to control for inherited linguistic similarity, as well as proposing a filter that separates languages long-established in a location close to their present one from those that have been recently displaced.

Future work is planned to make use of these linguistic similarity and temporal displacement variables to a greater extent and to address issues with the small number of languages for which environmental sampling areas are excessively large and/or represent artifacts of their specific spatial context. Additional future work includes the generation of global raster datasets representing the distribution of linguistic characteristics and the potential adoption of globally gridded historic climate data as an alternative to the point meteorological data currently used in the system. This alternative representation of language and climate characteristics will provide opportunities for the use of raster spatial statistical analyses (such as spatial principal components analysis) as an alternative to the non-spatial statistical analyses that have been performed to date. Finally, several datasets (elevation, weather station, specific humidity) included in the system include diagnostic and QA/QC data as part of their data model. Future work will endeavor to integrate these quality data values into the analytic workflow, providing a more robust interpretation of results.

## Data availability statement

Publicly available datasets were analyzed in this study. This data can be found as follows: The global linguistic dataset developed for and used in this project is available under the Creative Commons Attribution 4.0 license from Maddieson and Benedict (2023). Global linguistic data. Doi: https://doi.org/10.5281/zenodo.7992389. The analytic platform configuration and code is available under the Apache License 2.0 open-source license from Benedict and Maddieson (2023). Analytic platform and code for global linguistic analysis. Doi: https://doi.org/10.5281/zenodo.7992359. The interactive web application that hosts the language data used in this analysis is: Maddieson et al. (2013–2023) LAPSyD database https://lapsyd.huma-num.fr/lapsyd/.

## Author contributions

KB contribution was primarily in the collection and critical evaluation of climatic and environmental data and the establishment of a system for processing these data in relation to linguistic variables. IM provided data on language identity, linguistic variables, language locations, and affiliations. All authors participated in discussions concerning drawing boundaries around language areas and statistical analysis of results.

## Conflict of interest

The authors declare that the research was conducted in the absence of any commercial or financial relationships that could be construed as a potential conflict of interest.

## Publisher’s note

All claims expressed in this article are solely those of the authors and do not necessarily represent those of their affiliated organizations, or those of the publisher, the editors and the reviewers. Any product that may be evaluated in this article, or claim that may be made by its manufacturer, is not guaranteed or endorsed by the publisher.

## Footnotes

^1^One reviewer suggested using the age of first and subsequent splits estimated in analyses under the Automated Similarity Judgment Program (ASJP). We have doubts about both the validity of the linguistic data used (radically simplified transcriptions of a short wordlist with no validation of cognates) and the methods used to calibrate dating. Holman et al. (2011) cover an impressive amount of data but there are many puzzling results. For example, the age assigned to Proto-Yeniseian is later than that assigned to its daughter Awin-Pumpokol branch, the age for Malayo-Polynesian is younger than that for an assumed Eastern Malayo-Polynesian sub-branch of Austronesian, and the age for Chibchan is younger than that for its daughter branch Rama. These are among many details that would make it near-impossible to use this data for assigning language distances.
